# “Can do” versus “do do”: A Novel Concept to Better Understand Physical Functioning in Patients with Chronic Obstructive Pulmonary Disease

**DOI:** 10.3390/jcm8030340

**Published:** 2019-03-11

**Authors:** Eleonore H. Koolen, Hieronymus W. van Hees, Rob C. van Lummel, Richard Dekhuijzen, Remco S. Djamin, Martijn A. Spruit, Alex J. van ’t Hul

**Affiliations:** 1 Department of Pulmonary Diseases, Radboud University Medical Center, 6525 GA Nijmegen, The Netherlands; jeroen.vanhees@radboudumc.nl (H.W.v.H.); richard.dekhuijzen@radboudumc.nl (R.D.); alex.vanthul@radboudumc.nl (A.J.v.H.); 2 McRobert B.V., 2596 HN The Hague, The Netherlands; r.vanlummel@mcroberts.nl; 3 Department of Pulmonary Diseases, Amphia Hospital, 4819 EV Breda, The Netherlands; rdjamin@amphia.nl; 4 Department of Research and Education, CIRO+, Center of Expertise for Chronic Organ Failure, 6085 NM Horn, The Netherlands; martijnspruit@ciro-horn.nl; 5 Department of Respiratory Medicine, Maastricht University Medical Centre, NUTRIM School of Nutrition and Translational Research in Metabolism, 6229 HX Maastricht, The Netherlands; 6 REVAL—Rehabilitation Research Center, BIOMED—Biomedical Research Institute, Faculty of Rehabilitation Sciences, Hasselt University, 3590 BE Diepenbeek, Belgium

**Keywords:** chronic obstructive pulmonary disease, exercise, physical function, physical activity

## Abstract

Background: Physical capacity (PC) and physical activity (PA) represent associated but separate domains of physical function. It remains unknown whether this framework may support a better understanding of the impaired physical function in patients with chronic obstructive pulmonary disease (COPD). The current study had two aims: (1) to determine the distribution of patients with COPD over the PC-PA quadrants, and (2) to explore whether differences exist in clinical characteristics between these quadrants. Methods: In this retrospective study, PC was measured using the six-minute walk distance (6MWD), and PA was assessed with an accelerometer. Moreover, patients’ clinical characteristics were obtained. Patients were divided into the following quadrants: (I) low PC (6MWD <70% predicted), low PA, using a step-defined inactivity index (<5000 steps/day, ”can’t do, don’t do” quadrant); (II) preserved PC, low PA (“can do, don’t do” quadrant); (III) low PC, preserved PA (“can’t do, do do” quadrant); and (IV) preserved PC, preserved PA (“can do, do do” quadrant). Results: The distribution of the 662 COPD patients over the quadrants was as follows: “can’t do, don’t do”: 34%; “can do, don’t do”: 14%; “can’t do, do do”: 21%; and “can do, do do”: 31%. Statistically significant differences between quadrants were found for all clinical characteristics, except for educational levels. Conclusions: This study proves the applicability of the PC-PA quadrant concept in COPD. This concept serves as a pragmatic clinical tool, that may be useful in the understanding of the impaired physical functioning in COPD patients and therefore, may improve the selection of appropriate interventions to improve physical function.

## 1. Introduction

Impaired physical capacity (PC) and low-level daily physical activity (PA) are common features in people with chronic obstructive pulmonary disease (COPD) [1]. PC refers to the ability to perform physical activities and is generally quantified by exercise tests [2]. Daily PA may be defined as any bodily movement produced by skeletal muscles that results in energy expenditure beyond that of the resting state [3]. These days, the measurement of PA is performed with activity monitors rather than with questionnaires [4].

Over the last decade, an increasing number of studies have shown the clinical relevance of low-level daily PA in COPD patients, as it is associated with poor health status, increased healthcare utilization, and higher mortality risk. Moreover, these associations are independent of the degree of airway obstruction [1]. Therefore, improving PA is considered to be pivotal to the comprehensive management of COPD patients [5].

Exercise training is a common intervention used to improve physical function in COPD patients, either as a single intervention or as part of pulmonary rehabilitation [6]. Despite the significant positive impact of such interventions on PC [7,8,9,10], the improvements in PA are disappointingly incongruous [11,12] and seem to point to some discrepancies between changes in PC levels (“can do”) and changes in habitual PA levels (“do do”) [13]. These counterintuitive observations may, however, be less surprising given the identification of multiple determinants of PA in patients with COPD of which PC is an important determinant, but not the only one [14]. The need for further research in this field has been acknowledged both by the American Thoracic Society and the European Respiratory Society [1,15].

Recently, a conceptual framework was published in which PC and PA were viewed as associated but separate domains of physical function in the elderly, enabling individually tailored interventions [16]. We used this framework as a starting point for the development of a PC-PA quadrant concept, in which COPD patients could be subdivided along axes of what they physically “can do” (PC), as in an exercise test, and what they actually “do do” (PA), in their daily lives.

The hypothesis of the present study is that using this PC-PA quadrant concept enables identification of subgroups of COPD patients with different clinical characteristics that may contribute to the explanation of the discrepancy between their PC and PA. The current study sets out to (1) determine the distribution of COPD patients over the proposed PC-PA quadrant concept, and (2) explore whether, and to what extent, differences exist in clinical characteristics between the patients subdivided into mutually exclusive PC-PA quadrants.

## 2. Experimental Section

### 2.1. Study Design and Participants

In this retrospective study, participants were patients over 40 years of age, with relatively stable COPD [5], who underwent a comprehensive health status assessment as part of the usual COPD care in Amphia Hospital in Breda and Radboud University Medical Centre in Nijmegen (both in The Netherlands) between April 2013 and June 2017. According to the Dutch Standard of Care for COPD, general practitioners referred these patients to pulmonologists in a secondary care setting because the patients had persistent respiratory symptoms and/or limited activities of daily living and an unsatisfactory response to the medical treatment offered in primary care. Patients with a COPD exacerbation in the previous three months were excluded, as exacerbation-related symptoms and physical inactivity could have still been present during this period [17,18,19]. The Medical Ethical Committee of the Radboudumc approved this retrospective study, and because the participants were subjected to usual care (ref: 2016–2603), they considered that it did not fall within the remit of the Medical Research Involving Human Subjects Act (WMO). Therefore, the de-identified and pre-existing data of 662 patients were used for analyses.

### 2.2. Assessments

In all patients, a standardized, comprehensive health status assessment was completed, as described elsewhere [20]. PC and PA were the two main outcomes. PC was measured with a six-minute walk test (6MWT) and expressed as a percentage of the predicted value [21] using the reference equation of Troosters et al. [22]. As peak oxygen uptake during a 6MWT was comparable with values obtained during a symptom-limited cardiopulmonary exercise test, it seems fair to conclude that the 6MWT can be considered a test of PC [23]. In addition, the 6MWT had the advantage of being a self-paced exercise test and allowed for the inclusion of patients into this study who exhibited extremely low exercise tolerance [21]. PA was objectively assessed with either an uniaxial accelerometer (Digiwalker SW-200; Yamax Corporation, Tokyo, Japan [24]) or a triaxial accelerometer (DynaPort MoveMonitor, McRoberts, The Hague, The Netherlands) for seven consecutive days, and it is expressed as the average number of steps per day measured over at least four valid days [25]. In addition, various patient and health-status characteristics were systematically registered. These characteristics included the following: age (years); gender (male/female); body mass index (BMI, body weight in kg divided by height in squared meters, kg/m²); waist circumference (cm; male ≥94 cm or female ≥80 cm are at risk for cardiovascular comorbidity) [26]; Global Initiative for Chronic Obstructive Lung Disease (GOLD) classification (I–IV and A–D) [5]; pulmonary function (spirometry and flow-volume curve, using the Global Lung Initiative (GLI) equations) [27]; number of patients with frequent COPD exacerbations, defined as an acute worsening of respiratory symptoms that result in additional therapy [5], in the last 12 months (infrequent: <2 exacerbations per year/frequent: ≥2 exacerbations per year); smoking status (current/former-never); partner (yes/no); employment status (yes/no); and educational level according to Verhage’s classification (low/intermediate/high) [28]. Table 1 provides an overview of the health status questionnaires that were used.

### 2.3. Statistical Analysis

Descriptive statistics were used to summarize the data as medians (ranges) or frequencies (proportions), as appropriate. The patients were divided into mutually exclusive categories using the quadrant concept on the basis of their PC and PA: (I) low PC (6MWD <70% of the predicted value [21]) and low PA (using a step-defined inactivity index <5000 steps per day [37,38], “can’t do, don’t do” quadrant); (II) preserved PC, low PA (“can do, don’t do” quadrant); (III) low PC, preserved PA (“can’t do, do do” quadrant);(IV) preserved PC and preserved PA (“can do, do do” quadrant). In the absence of a validated minimum value for PC that would interfere with the normal ability to perform daily tasks, the PC threshold was calculated. By putting two standard deviations below the mean value of the non-COPD subjects, we knew that only about 2.5% of the non-COPD subjects had such abnormally low 6 min walk distances. The mean 6MWD in non-COPD control subjects (631 m) was set as 100% of the predicted value [22], and one standard deviation (93 m) matched 15% of the predicted value [22]. Therefore, the mean (100%) minus twice the standard deviation resulted in an arbitrary, but statistically reasonable cut off of 70% of the predicted value for PC. Furthermore, the threshold of a low PA was defined as <5000 steps per day. The continued use of <5000 steps per day as a step-defined sedentary lifestyle index for adults is appropriate for researchers and practitioners and for communicating with the general public [38] and has also been validated for COPD patients [37]. Differences between quadrants were tested with non-parametric tests, because the number of patients in the quadrants was not equal. Therefore, Kruskal–Wallis or Chi-square tests were used, including post-hoc analysis, as appropriate. The Pearson’s correlation coefficient was used to evaluate the association between PC and PA, and *p*-values below 0.05 were considered statistically significant. All statistical analyses were conducted using SPSS Version 22 (IBM Corp., Armonk, NY, USA).

## 3. Results

A total of 662 elderly patients with COPD were available for analyses. The majority were men (55%) who had a mild to very severe degree of airflow limitation and had a marked heterogeneity in their health status scores (Table 2). In brief, 53% of the COPD patients had a high symptom burden based on a CCQ score of ≥1.9 points and 50% based on a CAT score of ≥18 points [35]. Moreover, functional exercise performance (mean 6MWD: 68% predicted) and the level of physical activity (median steps per day: 5112) were abnormally low. There were no statistically significant differences between subgroups measured with the Digiwalker SW-200 or the Dynaport MoveMonitor with respect to PA (5328 ± 3664 versus 5700 ± 2897 steps per day; *p* = 0.146), PC (6MWD: 67 ± 15 versus 67 ± 15% predicted; *p* = 0.876), age (64 ± 10 versus 64 ± 9 years; *p* = 0.974), or the degree of airflow limitation (FEV1: 59 ± 20 versus 58 ± 18% predicted; *p* = 0.667).

**Figure 1:**
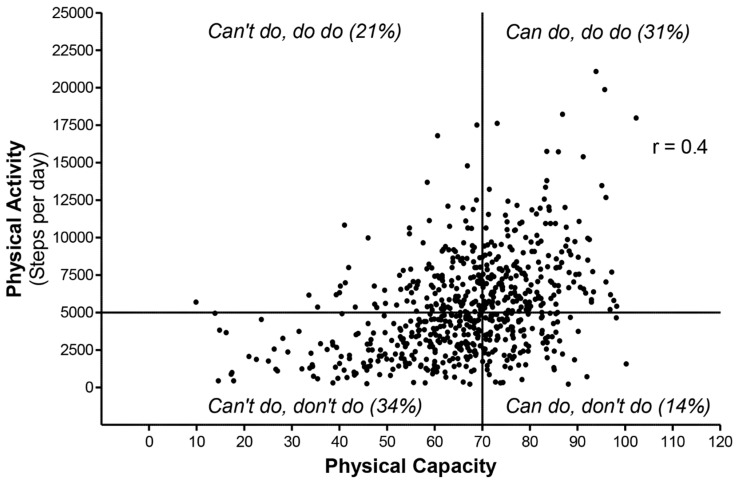
Graphical overview of the physical capacity-physical activity quadrant concept. 6MWD = six-minute walk distance; *r* = Pearson’s correlation coefficient; sample: *n* = 662 COPD patients.

The distribution of patients over the PC-PA quadrants was as follows: (1) “can’t do, don’t do”: 34%, (2) “can do, don’t do”: 14%, (3) “can’t do, do do”: 21%, and (4) “can do, do do”: 31% (Figure 1). The Pearson’s correlation coefficient between PC and PA was 0.4 (*p* < 0.001). Statistically significant differences between PC-PA quadrants were found for all the characteristics, except for the educational levels (Table 2).

## 4. Discussion

The main findings from this study are (1) the proposed PC-PA quadrant concept enables subdivision of patients with COPD into four exclusive subgroups with distinctive PC-PA values, and (2) these PC-PA-based quadrants are considerably different in multiple clinical characteristics.

PC and PA showed a low but significant correlation (*r* = 0.4; *p* < 0.001), which is in line with earlier studies and confirms that PC is just one determinant of PA [14,16]. Psychosocial and behavioral aspects are equally important for understanding and targeting low-level daily PA in individual patients [39]. The PC-PA quadrant concept enables identification of subgroups of COPD patients with definable treatable traits and may be useful in the stratification of appropriate non-pharmacological interventions aiming to improve physical function in future studies (i.e., pulmonary rehabilitation and PA coaching), as was suggested earlier in an editorial by Singh [40].

The patients in the “can’t do, don’t do” quadrant were mostly the patients with the highest disease burden, on the basis of the degree of pulmonary function impairment, comorbidities, exacerbation frequency, and symptom load, factors that are, not surprisingly, associated with the largest impact on overall health status. Because of the multiple and complex treatable traits in this subgroup, it identifies them as suitable candidates for a comprehensive pulmonary rehabilitation program [9]. To improve PA, it is suggested that the traditional approach to pulmonary rehabilitation with supervised high-intensity exercise training as the cornerstone may have little transfer-effect on an increase in PA [41]. Adaptations of such programs to turn improved PC into more active lifestyles seems feasible and results in higher PA [42].

The patients in the “can do, don’t do” quadrant only showed a trivial difference in PC compared to those in the “can do, do do” quadrant [21]. By contrast, the statistically significant difference in PA of 4201 (56%) fewer steps per day is huge and exceeds the assumed threshold of clinical relevance in PA for COPD patients by four times [43]. In the “can do, don’t do” quadrant, patients potentially have the ability to be active, but they “just” don’t do it. Although exercise training on top of promoting PA resulted in improved exercise capacity in patients with mild to moderate COPD, it did not translate into statistically significant enhanced daily PA [44]. Targeting behavioral change in order to increase PA could be the more appropriate management strategy in this subgroup but certainly will not be an easy task [45]. To address behavioral change at the individual level, all personal barriers and enablers that may hinder or facilitate PA engagement must be considered in future studies [46]. Also, observations of the “can do, don’t do” quadrant showed the highest BMI and the largest percentage of patients with a high waist circumference as compared to the other quadrants. Weight reducing measures may be important to improve PA and might also positively affect the risk of obesity related comorbidities [47]. Then again, a lack of PA may also have caused the high BMI which may improve by becoming physically more active [48]. Finally, a remarkable finding in this “can do, don’t do” quadrant is the relatively low proportion of patients with frequent exacerbations, especially compared with the “can’t do, don’t do” quadrant (10% versus 35%). An earlier study showed a relationship between PA and exacerbation risk [49], which suggests that especially the combination of a low PC and low PA may predispose patients to repeated exacerbations.

In the “can’t do, do do” quadrant, the median number of steps per day was 4197 (148%) higher than that of the “can’t do, don’t do” quadrant. However, despite the fact that the allocation criterion for PC was the same for these quadrants, the difference in 6MWD of 72 m, although not statistically significant, exceeds the threshold of clinical relevance. This unanticipated difference in PC between these two quadrants might, at least to some extent, be accountable for the marked difference in PA. Other clinical characteristics that may explain the higher PA in this subgroup are the younger age, the possibly related larger proportion of patients with a job, and the greater proportion of patients with a partner. The latter observation seems to be consistent with the finding that social support and objective indices of support by spouses, friends, or work colleagues are important enablers for improving PA levels generally [50], but also in COPD patients [39]. Furthermore, it is imperative to understand all the perceived psychosocial barriers and enablers of engagement in PA [39]. Therefore, further qualitative research into psychosocial barriers and enablers in this population is required to eventually develop interventions aimed at reducing perceived barriers while optimizing enablers. Finally, the patients in the “can do, do do” quadrant, both with preserved PC and PA, were actually the patients with the best overall outcomes. Obviously, there were reasons to refer these patients to pulmonologists, indicating the presence of clinically relevant symptoms and/or functional limitations. Within this quadrant, treatment interventions should not be primarily focused on the level of PC or PA, but on other treatable traits regarding their burden of disease, such as self-management support, medication adherence, or cognitive behavioral therapy. Taking the individual traits into account applies to this quadrant, and to the other three quadrants, in order to provide the best personalized care [51].

This study has several strengths. A large, real-life sample of COPD patients was recruited, who were referred by their general practitioner to the pulmonologist. This supports the external validity and generalizability of the findings in this study. Interestingly, high symptom burden [35], physical inactivity, and physical deconditioning were identified in a significant proportion of patients. This raises the question as to why this group of patients was referred to the pulmonologist at an apparently random stage. Also, an important point is that patients were comprehensively assessed, allowing for comparison between the quadrants using multiple patient characteristics. There are also some methodological considerations. Obviously, a change in PC and/or PA cut off points will redistribute the patients, especially in patients close to the cut points. The aim of the present study was, however, not to determine the clinically relevant thresholds of PC and PA precisely, but rather to demonstrate proof of the PC-PA concept in COPD patients.

## 5. Future Studies and Conclusions

This study provides proof of the PC-PA quadrant concept in COPD patients. Using this concept, it turned out to be possible to subdivide patients into exclusive quadrants with distinctive PC-PA relations. Obviously, future studies have to determine the extent to which PC-PA quadrants are useful in optimizing personalized medicine of patients with COPD, and their role in helping to better understand the association between low PA and/or PC and hospitalization risk. For current clinical practice, the PC-PA quadrant concept may already serve as a pragmatic clinical tool, which may be useful in the interpretation of the physical functioning of patients with COPD.

## Figures and Tables

**Table 1:** Overview questionnaires.

Questionnaire	Quantifies	Range	Interpretation
Charlson Comorbidity Index (CCI) [29]	Predict one-year mortality based on the presence of comorbidities	0–46	Higher scores indicate a higher mortality risk
Medical Research Council (MRC) dyspnea scale [30]	The degree of activity-related breathlessness	1–5	Higher scores indicate higher impact of dyspnea
Checklist Individual Strength fatigue domain (CIS) [31]	The degree of general fatigue	8–56	Higher scores indicate more fatigue
COPD Assessment Test (CAT) [32,33]	Burden of disease	0–40	Higher scores indicate higher burden of disease
COPD Clinical Questionnaire (CCQ) [34,35]	Burden of disease	0–6	Higher scores indicate higher burden of disease
Marshall Questionnaire [36]	Self-reported PA	0–8	Higher scores indicate higher level of self-reported PA

**Table 2:** Characteristics.

Attribute	Total Sample *N* = 662	“Can’t do, don’t do” *N* = 223; 34%	“Can do, don’t do” *N* = 96; 14%	“Can’t do, do do” *N* = 140; 21%	“Can do, do do” *N* = 203; 31%	Sig.
Age ^a^, years	63 (41–87)	67 (42–86) ^†,‡^	67 (46–87) ^†,‡^	59 (41–83)	62 (41–82)	<0.001
Female ^b^, %	45	41	49	40	52	<0.05
BMI ^a^, kg/m²	25.3 (14.1–51.6)	25.6 (14.1–51.6) *^,†^	27.6 (18.7–47.1) ^†,‡^	23.6 (15.5–36.6)^‡^	25.1 (16.5–40.4)	<0.001
High WC ^b^, %	78	76 *	90 ^†^	70	80	<0.01
GOLD I/II/III/IV ^b^, %	14/51/31/4	9/43/41/8 *^,‡^	16/58/25/1	11/55/30/4	20/53/25/2	<0.001
GOLD A/B/C/D ^b^, %	13/34/8/46	3/27/7/62 *^,†,‡^	12/51/9/28	12/37/11/41	23/30/7/40	<0.001
FEV1 % pred. ^a^	56 (14–116)	51 (14–111) *^,†,‡^	61 (28–109)	56 (21–07)	60 (23–116)	<0.001
FVC % pred. ^a^	93 (42–151)	89 (42–135) *^,‡^	94 (50–150)	91 (54–131) ^‡^	97 (49–151)	<0.001
FEV1/FVC ratio ^a^	0.48 (0.2–0.7)	0.45 (0.2–0.7) *^,‡^	0.51 (0.2–0.7)	0.48 (0.2–0.7)	0.51 (0.2–0.7)	<0.001
CCI ^a^, points	3 (0–9)	3 (0–9) ^‡^	3 (0–7) ^‡^	2 (0–6)	2 (0–6)	<0.01
Frequent exacerbator. ^b^, %	26	35 ^*^	10 ^†^	26	23	<0.001
Current smokers ^b^, %	47	55 ^‡^	48	54 ^‡^	35	<0.001
Partnered ^b^, %	71	64 ^‡^	69	76	77	<0.05
Employed ^b^, %	32	17 ^†,‡^	28	43	44	<0.001
Low/Intermediate/High Educational level ^b^, %	16/65/18	15/64/21	18/68/14	14/66/20	19/64/17	0.289
CAT ^a^, points	18 (0–40)	21 (1–40) ^†,‡^	18 (2–37) ^‡^	17 (0–37)	14 (1–35)	<0.001
MRC grade ^a^	2 (0–5)	3 (0–5) *^,†,‡^	2 (0–5) ^‡^	2 (0–5) ^‡^	2 (0–5)	<0.001
CIS Fatigue ^a^, points	37 (8–56)	43 (8–56) ^†,‡^	36 (13–56) ^‡^	36 (8–56)	33 (8–56)	<0.001
CCQ ^a^, points	2.0 (0.1–5.8)	2.7 (0.2–5.8) *^,†,‡^	2.0 (0.3–4.8)	1.8 (0.1–5.1) ^‡^	1.5 (0.1–4.7)	<0.001
Marshall Quest. ^b^ ≥4 points, %	45	31 ^‡^	33 ^‡^	45	64	<0.01
6MWD ^a^, m	440 (76–805)	351 (100–558) *^,†,‡^	485 (317–660) ^†,‡^	423 (76–550) ^‡^	512 (339–805)	<0.001
6MWD% pred ^a^	68 (10–102)	58 (15–70) *^,‡^	76 (70–100) ^†^	63 (10–70) ^‡^	79 (70–102)	<0.001
Steps per day ^a^	5112 (333–21191)	2838 (345–4998) ^†,‡^	3355 (333–4961) ^†,‡^	7035 (5016–17621)	7556 (5000–21191)	<0.001

BMI = Body Mass Index; High WC = High Waist Circumferences (Male >94 cm, Female >80 cm); GOLD = Global Initiative for Chronic Obstructive Lung Disease; FEV1% pred. = Forced Expiratory Volume in one second percentage predicted; FVC% pred. = Forced Vital Capacity percentage predicted; CCI = Charlson Comorbidity Index; CAT = COPD Assessment Test; MRC dyspnoea scale = Medical Research Council dyspnoea scale; CIS = Checklist Individual Strength; CCQ = COPD Clinical Questionnaire; 6MWD = Six-Minute Walking Distance; 6MWD% pred = Six-Minute Walking Distance percentage predicted. Missing sample data per category = *n* (%): Waist circumferences = 42 (6); GOLD (A-D) = 116 (18); CCI = 328 (58); Exacerbations <12 months = 123 (19); Current smoking status = 23 (3); Partner = 37 (6); Employment = 47 (7); Educational level = 55(8); CAT = 138 (21); CCQ total = 62(9); MRC dyspnoea scale = 70 (11); Marshall questionnaire = 464 (70); CIS = 21 (3) ^a^: Continuous variable: Median (Range) ^b^: Categorical variable: Proportion (%) *: *p* < 0.05 versus “can do, don’t do” ^†^: *p* < 0.05 versus “can’t do, do do” ^‡^: *p* < 0.05 versus “can do, do do”.
